# Perspective or Spectacle? Teaching thoracic aortic anatomy in a mixed reality assisted educational approach– a two-armed randomized pilot study

**DOI:** 10.1007/s00423-024-03463-0

**Published:** 2024-09-09

**Authors:** Lea Zimmer, Johannes Hatzl, Christian Uhl, Samuel Kilian, Moritz S. Bischoff, Dittmar Böckler, Katrin Meisenbacher

**Affiliations:** 1 Department of Vascular and Endovascular Surgery, University of Heidelberg, Heidelberg, Germany; 2 Department of Vascular Surgery, University Hospital RWTH Aachen, 52074 Aachen, Germany; 3 Institute of Medical Biometry, University of Heidelberg, Heidelberg, Germany

**Keywords:** Mixed reality, Thoracic aorta, Vascular surgery, Virtual reality, Surgical education

## Abstract

**Purpose:**

Anatomical understanding is an important basis for medical teaching, especially in a surgical context. The interpretation of complex vascular structures via two-dimensional visualization can yet be difficult, particularly for students. The objective of this study was to investigate the feasibility of an MxR-assisted educational approach in vascular surgery undergraduate education, comparing an MxR-based teaching-intervention with CT-based material for learning and understanding the vascular morphology of the thoracic aorta.

**Methods:**

In a prospective randomized controlled trial learning success and diagnostic skills following an MxR- vs. a CT-based intervention was investigated in 120 thoracic aortic visualizations. Secondary outcomes were motivation, system-usability as well as workload/satisfaction. Motivational factors and training-experience were also assessed. Twelve students (7 females; mean age: 23 years) were randomized into two groups undergoing educational intervention with MxR or CT.

**Results:**

Evaluation of learning success showed a mean improvement of 1.17 points (max.score: 10; 95%CI: 0.36–1.97). The MxR-group has improved by a mean of 1.33 [95% CI: 0.16–2.51], against 1.0 points [95% CI: -0.71- 2.71] in the CT-group. Regarding diagnostic skills, both groups performed equally (CT-group: 58.25 ± 7.86 vs. MxR-group:58.5 ± 6.60; max. score 92.0). 11/12 participants were convinced that MxR facilitated learning of vascular morphologies. The usability of the MxR-system was rated positively, and the perceived workload was low.

**Conclusion:**

MxR-systems can be a valuable addition to vascular surgery education. Further evaluation of the technology in larger teaching situations are required. Especially regarding the acquisition of practical skills, the use of MxR-systems offers interesting application possibilities in surgical education.

## Introduction

Year after year, the trajectory of medical-technical advancements is characterized by a continuous refinement and revolutionary transformation, manifesting in heightened efficiency and specialization. However, to maintain a truly forward-looking perspective, it would be myopic to confine innovation solely within the domain of clinical research. Ensuring the cultivation of adept clinical practice among future physicians necessitates the concurrent integration of technological progress into medical curricula. This imperative extends equivalently to the augmentation of conventional pedagogical approaches. Commencing with fundamental principles, the establishment of a comprehensive understanding of human anatomy constitutes a cornerstone pivotal to both medical education and advanced surgical training. Implementing anatomical models, and later three-dimensional (3D)-printing-methods in anatomy education, offered options to understand and teach topography in a realistic 3D-environment, rather than relying solely on two-dimensional (2D) anatomy atlases [1]. Clearly, studying anatomy from cadaver dissection is an excellent way to develop a first understanding for gross anatomy [2]. Due to high costs, limited availability, elaborate infrastructure, and time effort for the cadaver dissection, and – not least – ethical concerns – supplementary methods for future anatomical curricula are needed. Understanding and interpreting complex vascular structures represents a major challenge for both students and inexperienced physicians. While 2-D-imaging-techniques such as computed tomography (CT) are already widely established during anatomical education [3–6], apparently – and equally to traditional anatomic atlas imaging – stereoscopic perception is not provided. Therefore, 3-D-visualization tools have been proposed to be more suitable for this purpose [7–9].

In recent years, applications of “extended reality” (XR) have found their way into the medical field and partially into medical education as well. [10]. XR is used as an umbrella term to address all immersive technologies including augmented reality (AR), virtual reality (VR), mixed reality (MxR) and those yet to be discovered. In AR, virtual information and objects are overlaid on the real world, whereas in VR the user fully immerses into a simulated environment. In MxR, we find the merging of a real-world environment and a computer-generated one. In this reality, real-world- and virtual objects co-exist and interact with each other [11]. The perception of a user’s physical environment is thus enhanced with an artificial computer-generated perception, enabling a realistic interaction via a 3-D virtual object. For the practical implementation of MxR, the user is wearning a head-mounted display (HMD). Software processing projects any digital information (e.g. the data from a CT-scan) into the user’s field of vision, appearing as an object showing a 3-D-reconstruction of the given digital information. In other fields, such as the gaming or the marketing industry, these technologies are already successfully implemented. In medicine, however, they have mostly been the subject of research and have not yet been introduced to clinical practice [12]. So far, only a few randomized studies have evaluated the use of MxR technology in medical student education [13]. This pilot study’s objective is the feasibility of an MxR-assisted educational approach in vascular surgery undergraduate education, aiming at learning and understanding anatomical features and vascular morphologies of the thoracic aorta and its branches in comparison to CT-based training material. In a randomized setting, the learning success of students with different teaching material will be investigated. In addition, the study will address several complementary questions such as the usability of the MxR-system, the student’s perceived workload and motivational factors.

## Methods

### Study design and participants

The underlying study represents a prospective, randomized controlled trial. The scheduled course was divided into three sections: preparation, training and intervention. The study was conducted at the Department of Vascular and Endovascular Surgery of the University Hospital Heidelberg, Germany. Twelve medical students (7 females, mean age 23 years) of the Ruprecht-Karls-University Heidelberg have been recruited on a voluntary basis. Students had to have completed their pre-clinical education to participate, therefore all of them have been previously exposed to the general anatomy curriculum. The pilot study was conducted within the elective surgical track “modern surgery – innovation, research and technology”, comprising a vascular surgical module called “knives, wires and imaging – applied vascular surgery”. Ethical approval was obtained from the Institutional Ethical Review Board of the University of Heidelberg (S-488/2023). Written informed consent was provided from all participants.

#### Preparation and training

**Fig. 1:**
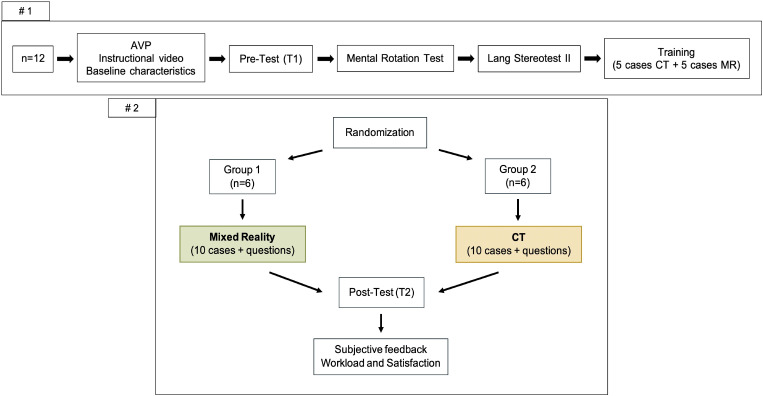
Study design and general course description. Upper image section: preparation and training; Bottom image section: randomization and intervention. AVP: audio-visual presentation; CT: computer tomography; n = Numbers

Before randomization, all participants followed the same preparation and training. During those, students were instructed to watch a 5-minute audio-visual presentation (AVP), discussing the anatomical basics of the vascular system, focusing on the thoracic aorta. The content of this video was compiled by a board-certified vascular surgeon. Additionally, the students had to watch an instructional 5-minutes video on how to handle the proposed MxR-system. For the following individual training, a set of 5 different anonymized CT-scans as well as 5 different MxR-objects were presented to the students. For this purpose, different morphologies of the thoracic aorta and its branches were selected, including as well healthy aortic conditions as anatomic variants and pathologies. The students were asked to analyze and explain what they see on each scan/object. A time limit of 5 min was set for each case, and an observer gave the students brief feedback after each case. This training session lasted about 90 min (Fig. 1, #1).

#### Randomization and intervention

After the first two parts, students were randomized into two balanced study groups using a lottery method (Fig. 1, #2). While participants randomized to the control group (*n* = 6) were confronted with 10 different CT-scans showing various morphologies of the thoracic aorta and its branches, participants in the intervention group (*n* = 6) were confronted with the same morphologies in terms of a 3-D-object using the MxR-system. Again, a time limit of 5 min was set for each case, during which the students were asked to review the visualized image and to provide their answers on a questionnaire. The intervention took place around 10 days after the training and pre-test part and again lasted about 90 min. To improve standardization, both training and intervention were monitored by the same observer throughout the whole study.

### Endpoints and Assessment

The primary outcome was the learning success and the diagnostic skills following intervention, thereby comparing the CT- vs. MxR-based intervention. Secondary outcomes were motivation, usability of the system as well as perceived workload and satisfaction.

#### Quantitative assessment

**Fig. 2:**
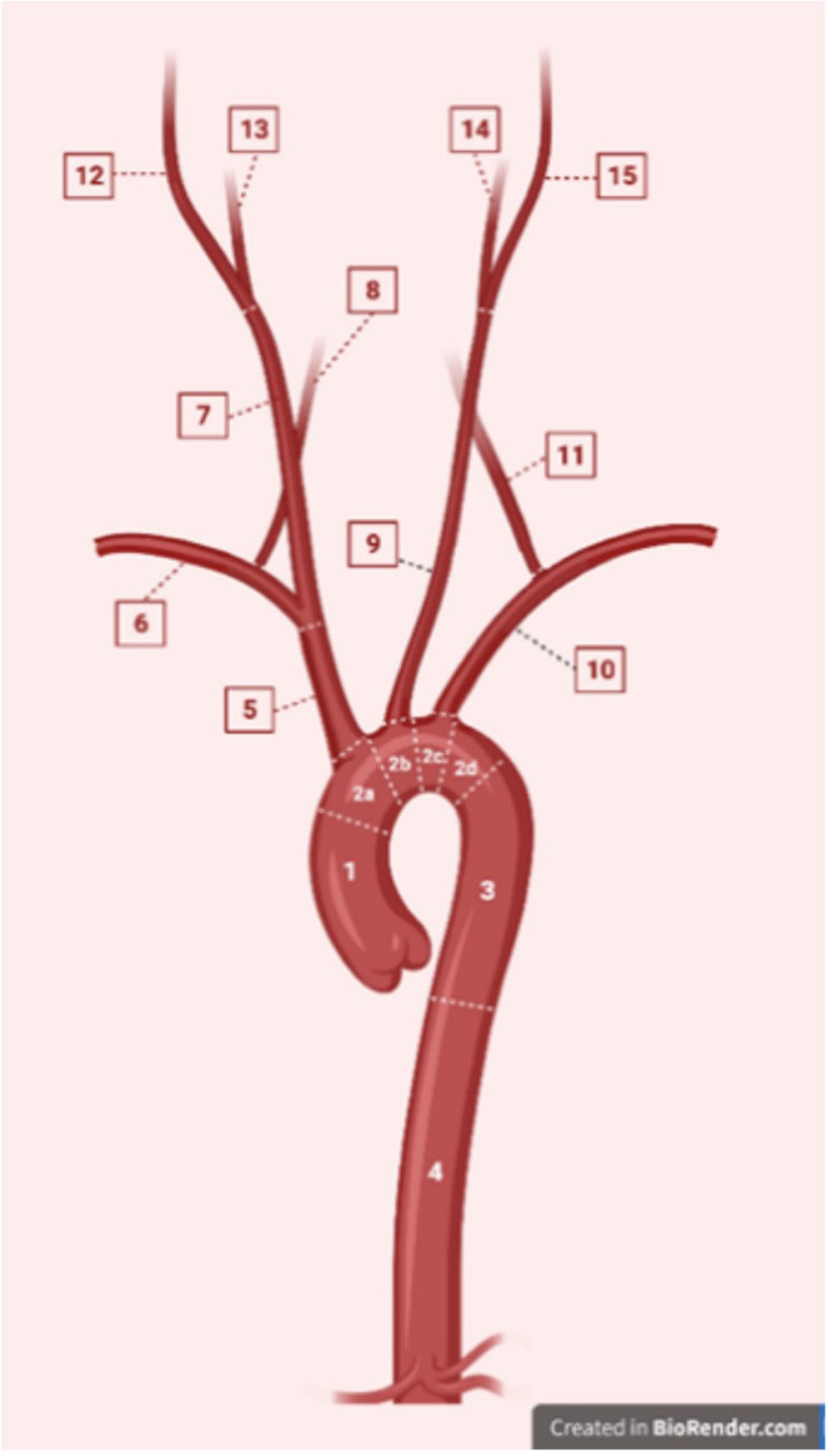
Illustration scoring anatomy, localization and morphology during intervention. During intervention, the participants had to assess whether the respective presented aortic anatomy was physiological, pathological, abnormal, or showed a post-operative condition. The task was to describe this morphology as well as possible and to name the relevant section using the demonstrated schematic illustration. (max. score: 92 points, time limit per aorta: 5 min)

During preparation, demographics and baseline knowledge were collected. Theoretical and image-based multiple choice (MC) questions focusing on the topography of the thoracic aortic system were used to assess learning success (T1: evaluation of student’s prior knowledge of topographic anatomy of the thoracic aorta; T2: after the intervention; 10 questions per test, maximum score: 10 points). Diagnostic skills were additionally tested in ten thoracic aortic visualizations per participants. Using a specifically designed illustration (Fig. 2) anatomic conditions (physiological condition, one/more pathological conditions, one/more anomalies, previously operated aorta; maximum score: 60 points), localization according to the correct aortic/aortic branch segment (maximum score: 16 points) and morphology (i.e. describing the morphological conditions and name it correctly; maximum score: 16 points) had to be evaluated, respectively. The answers were subsequently scored by the observers following a given scoring system, a maximum of 92 points could be reached. In addition, all participants were asked to perform a mental rotation test to quantify their spatial ability (max. score: 24 points) [14, 15]. The Lang Stereotest II (Lang Stereotest, Forch, Switzerland) was carried out to detect whether stereoscopic vision was intact or not, since impairments in stereovision highly influence the ability to perceive virtual objects as three-dimensional in a MxR-environment [16].

#### Qualitative assessment

Perceived workload and satisfaction were assessed via the NASA Task load Index (NASA-TLX). This validated assessment tool scores six domains (mental demand, physical demand, performance, effort, and frustration) ranging from 0 (very low) to 100 (very high) in steps of five [17, 18]. For evaluation of the usability of the applied teaching instrument, the Standard System Usability Scale (SUS) was used. A score of 100 indicates high usability [19]. To further evaluate motivational aspects, students were given a self-designed questionnaire. A 4-point Likert scale was used to avoid a neutral position. All data was collected in pseudonymous form. Assessment performed during this study did not influence the students’ final grade.

### Learning environment, hard- and software

The course was conducted in a consultation room in the surgical clinic, suiting for 2 students, equipped with a computer and a two-dimensional monitor with access to standard digital imaging and communications in Medicine (DICOM) images as well as the picture archiving and communication system (PACS) of our institution. All images were shown after anonymization. For the visualization of objects in the MxR-environment, an MxR-workstation, consisting of a personal computer (PC) running the Elements Viewer software (Brainlab AG, Munich, Germany) as well as a HMD (Magic Leap1, Magic Leap, Florida, USA). The MxR-workflow has been previously described [20]. During training and intervention in the MxR-group, the participant as well as the observer wore an HMD. In order to interact with the virtual object, the participant was additionally equipped with a controller. For testing and evaluation, every participant was equipped with a digital device (IPad Pro 11, Apple Inc., California, USA).

### Statistical analysis

The collected data were analyzed using MS Excel Version 16.77.1 (Microsoft Corporation Washington, USA) and GraphPad Prism (Version 10, Graphpad Software, Inc.) Descriptive data evaluation was performed. Data are presented as absolute/relative frequencies or mean and standard deviation (SD). For group comparisons and comparisons of time points the Mann Whitney U test and the Wilcoxon-signed rank test were used. Multiple regression analysis was applied for estimation of any relation between baseline characteristics and learning success. All p-values are of descriptive nature.

## Results

### Baseline characteristics

A total of 12 students participated in this study. All of them completed all three parts of the study, so no dropout is to report. The participants´ demographics are shown in Table 1. None of the surveyed characteristics revealed an unbalanced distribution among the two study groups. All of them had either started or already finished the surgical part of their training, so at least some vascular-/surgical knowledge was present. Only one student mentioned related knowledge of the above and since him/her being in the control group no further importance was attached to the topic.

**Table 1:** Participants´ demographics

Characteristics *n* (%)	Overall (*n* = 12)	MxR (*n* = 6)	CTA (*n* = 6)	*p*-value
Gender				
*Female*	7 (58.33%)	4	3	
*Male*	5 (41.67%)	2	3	
Age (mean)^(1)^	23 years	22	23	
Educational level				
4th year	7 (58.33%)	4	3	
5th year	5 (41.67%)	2	3	
Surgical undergrad education				
*Started*	6 (50%)	3	3	
*Completed*	6 (50%)	3	3	
Previous experience with MR/VR ^(1)^	1 (9.09%)	0	1	
Video game experience^(1)^				
*No*	5 (45.45%)	3	2	
*Some*	3 (27.3%)	2	1	
*Yes*	3 (27.3%)	0	3	
Knowledge of programming language^(1)^	2 (18.2%)	1	1	
Active on social media^(1)^				
*No*	3 (27.3%)	1	2	
*Sometimes*	6 (54.55%)	3	3	
*Often*	2 (18.2%)	1	1	
Different job/studies before medical school^(1)^	1 (9.09%)	0	1	
Experience in vascular surgery^(1)^	1 (internship)	0	1	
Mental rotation test (mean ± SD)	17.92 ± 5.03	19 ± 4.19	16.83 ± 5.95	*0.385*
Intact stereovision (Lang II stereotest)	12 (100%)	6	6	

Data are given as absolute/relative numbers and mean ± standard deviation (SD), ^(1)^*n* = 11 due to uncompleted questionnaire

### Learning success

**Fig. 3:**
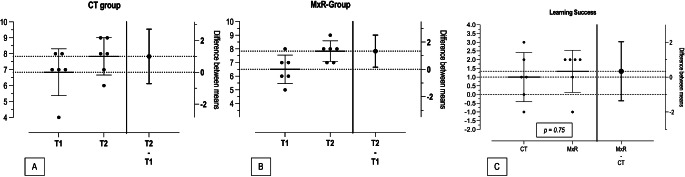
Estimation Plots of learning success. (**A**) depicts the improvement between T1 and T2 in the CT-group, (**B**) in the MxR-group. (**C**) shows the mean improvement in both groups (T2-T1)

Overall, the students scored a mean of 6.67 ± 1.23 points in the pre-test (T1) versus 7.83 ± 0.94 in the post-test (T2), showing a mean improvement of 1.17 points (95% CI: 0.36–1.97; *p* = 0.0156). The MxR-group showed a mean improvement of 1.33 [95% CI: 0.16–2.51] points, against 1.0 [95% CI: -0.71- 2.71] in the CT-group (Table 2; Fig. 3). Subanalysis of group-differences with respect to demographic characteristics such as educational level, surgical training, or gaming experience revealed no significant differences in improvement between T1 to T2. Considering the role of gender on learning success, female participants showed an overall mean improvement of 1.43 points [95% CI: 0.25–2.61] against 0.8 points [95% CI: -0.82 -2.42] in the male cohort. This difference was greater in the CT-group (mean 2.0 points of improvement in female vs. 0.0 points in male) and reverse in the MxR-group (1.0 points of improvement in female vs. 2.0 in male) (Table 2). Multiple linear regression for the outcome variable “learning success” (T1-T2) indicated “gender” and “educational level” as possible predictors (Table 3).

**Table 2:** Learning success

Group	T1	T2	MD [95% CI]	*p*-value
**Overall (** ***n*** ** = 12)**	**6.67 ± 1.23**	**7.83 ± 0.94**	**1.17 [0.36–1.97]**	**0.0156**
*Male (n = 5)*	*6.8 ± 1.30*	*7.6 ± 1.14*	*0.8 [-0.82 -2.42]*	*0.375*
*Female (n = 7)*	*6.57 ± 1.27*	*8.0 ± 0.82*	*1.43 [0.25–2.61]*	*0.0625*
**CT group (** ***n*** ** = 6)**	**6.83 ± 1.47**	**7.83 ± 1.17**	**1.0 [-0.71- 2.71]**	**0.250**
*Male (n = 3)*	*7.66 ± 0.58*	*7.66 ± 1.53*	*0 [-2.48–2.48]*	*> 0.99*
*Female (n = 3)*	*6 ± 1.73*	*8 ± 1*	*2.0 [-0.48-4.48]*	*0.250*
**MxR group (** ***n*** ** = 6)**	**6.5 ± 1.05**	**7.83 ± 0.75**	**1.33 [0.16–2.51]**	**0.0938**
*Male (n = 2)*	*5.5 ± 0.71*	*7.5 ± 0.71*	*2 [-1.04- 5.04]*	*n.a.*
*Female (n = 4)*	*7.0 ± 0.82*	*8 ± 0.82*	*1 [-1.25–3.25]*	*0.375*

T 1/T2: Given as mean ± standard deviation; CI: confidence interval; MD: mean of differences (T2-T1); n.a.: not applicable

**Table 3:** Multiple linear regression for the outcome variable learning success T1-T2

Variable	estimate	95% CI	*P* value
**Intercept**	-0.8361	-3.745–2.073	0.4932
Gender [female]	**1.549**	-1.284–4.383	0.2189
group [CT]	0.2596	-1.852–2.372	0.7648
gaming experience [no]	0.6120	-1.994–3.218	0.5724
social media [yes]	-0.1831	-2.996–2.630	0.8737
educational level/year	**1.440**	-1.116–3.996	0.2072

CT: computer tomography; CI: confidence interval

### Imaging evaluation

In assessment of thoracic aortic conditions regarding anatomy, localization of potential anomalies/pathologies and description of morphology, both groups performed equally with a total score of 58.25 ± 7.86 in the CT-group vs. 58.50 ± 6.60 in the MxR-group (max. score: 92.0), with a mean difference of 0.25 [95% CI: -4.345–4.845] between the groups. In the subscales, the MxR-group showed a tendency to better performance regarding anatomical tasks (CT-group: 47.83 ± 3.82 vs. MxR-group: 48.67 ± 3.20; mean difference: 0.83 [95% CI: -0.97–2.641], *p* = 0.675). (Table 4). In contrast, men seemed to perform slightly better than women (59.70 ± 9.060 vs. 57.43 ± 5.533; mean difference: 2.27 [95% CI: -7.064-11.61]; *p* = 0.755).

**Table 4:** Imaging evaluation

	Overall (*n* = 12)	CT group (*n* = 6)	MxR group (*n* = 6)	*p*-value	MD [95% CI]
Anatomy (max. 60 P)	48.25 ± 3.38	47.83 ± 3.82	48.67 ± 3.20	*0.675*	0.83 [-0.97–2.641]
Localization (max. 16 P)	2.86 ± 3.14	3.17 ± 3.23	2.58 ± 3.29	*0.368*	-0.58 [-6.258 -5.092]
Morphology (max. 16 P)	7.25 ± 2.19	7.25 ± 2.04	7.25 ± 2.53	*0.987*	0.0 [-1.787- 1.787]
**Total (max. 92 P)**	**58.38 ± 6.92**	**58.25 ± 7.86**	**58.50 ± 6.60**	***> 0.99***	**0.25 [-4.345–4.845]**

All values are given as mean ± standard deviation; CI: confidence interval; MD: mean of differences (T2-T1); P: points

### Evaluation of the MxR technology

Usability of the MxR-system was scored at a mean of 85.0 ± 6.93 points (maximum score: 100 = high usability) (Table 5). With respect to workload, in the NASA-TLX, the MxR-system was associated with a higher level of mental demand compared to the physical demand. The effort to accomplish the requested performance was rated low, as was the level of frustration (Table 6).

**Table 5:** Evaluation of the MxR-system using the standardized system usability scale (SUS)

	Strongly disagree	Disagree	Neutral	Agree	Strongly agree
I think that I would like to use this system frequently	-	-	16.7%	33.3%	50.0%
I found this system unnecessarily complex	66.7%	33.3%	-	-	-
I thought the system was easy to use	-	-	8.3%	41.7%	50.0%
I think that I would need the support of a technical person to be able to use this system	25.0%	50.0%	16.7%	8.3%	-
I found the various functions in this system were well integrated	-	16.7%	8.3%	58.3%	16.7%
I thought there was too much inconsistency in this system	50.0%	25.0%	25.0%	-	-
I would imagine that most people would learn to use this system very quickly	-	-	8.3%	33.3%	58.3%
I found the system very cumbersome to use	75.0%	25.0%	-	-	-
I felt very confident using the system	-	-	-	58.3%	41.7%
I need to learn a lot of things before I could get going with this system	33.3%	58.3%	8.3%	-	-

Usability of the MxR-system was scored at a mean of 85.0 ± 6.93 points. A score of 100 indicates high usability. Values are given in percentage or absolute values

**Table 6:** NASA TLX

	Overall	CT group	MxR group	*p*-value
Mental demand	56.67 ± 17.75	51.67 ± 23.17	61.67 ± 9.83	*0.560*
Physical demand	32.50 ± 17.65	26.67 ± 13.66	38.33 ± 20.41	*0.292*
Temporal demand	37.50 ± 12.88	38.33 ± 11.69	36.67 ± 15.06	*> 0.999*
Performance	68.33 ± 18.01	71.67 ± 16.02	65.0 ± 20.74	*0.556*
Effort	38.33 ± 11.15	41.67 ± 11.69	35.0 ± 10.49	*0.461*
Frustration	24.17 ± 18.32	23.33 ± 23.38	25.0 ± 13.78	*0.471*

Given as mean ± standard deviation; NASA-TLX: NASA task load index (0 = very low, 100 = very high)

**Fig. 4:**
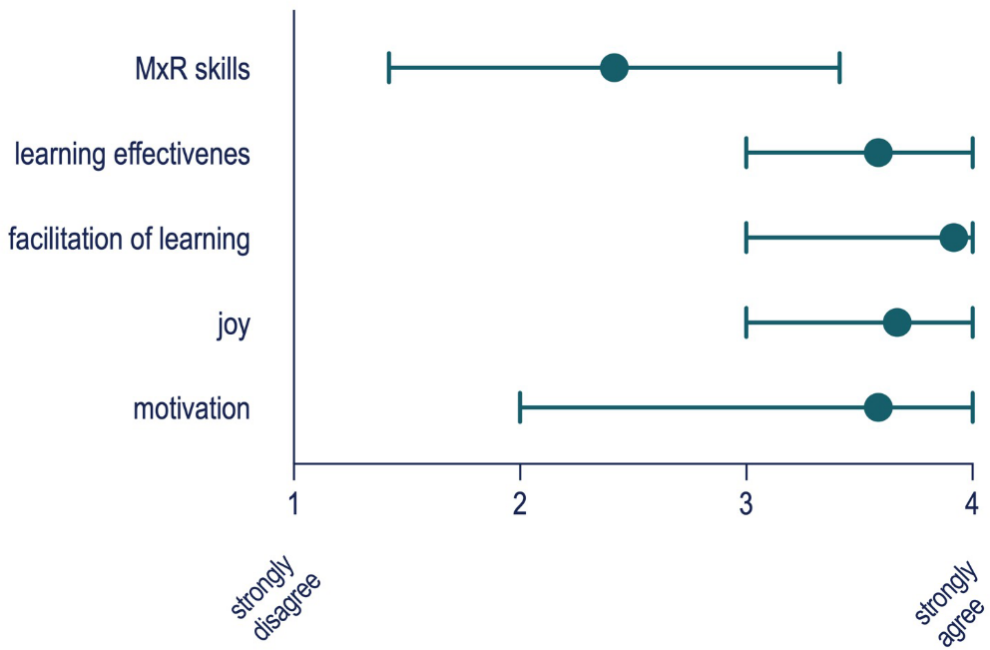
Subjective feedback on the MxR-System with respect to motivational aspects. Questions were: 1. How do you rate your skills in the field of MxR?; 2: I have learned a lot through the use of MxR; 3: I can imagine that MxR makes it easier to learn vascular anatomy.; 4: I enjoy learning new things with MxR more than with traditional teaching methods.; 5: I am more motivated to learn new medical knowledge with MxR than with traditional teaching methods. Rating was performed using a 4-Point Likert scale; 1: strongly disagree; 4: strongly agree

Motivational aspects regarding the MxR-system were rated high (mean 3.58 ± 0.67) as was facilitation of learning (mean 3.92 ± 0.28, 4-point Likert scale, 4 = strongly agree) (Fig. 4). Participants agreed on the exceedingly positive effects of MR systems onto motivational aspects.

## Discussion

This study shows that an MxR-system is suitable in teaching anatomical knowledge and vascular morphology of the thoracic aorta. Summarizing the main findings, learning success was recorded in both, the MxR- and the control (CT) group, indicating the general effectiveness of this teaching approach. Although there was no significant difference in learning success between both groups, participants randomized to the MxR-group improved slightly more, suggesting a potential advantage of using MxR as a visualization tool to acquire anatomical knowledge. Both imaging methods are appropriate for assessing complex vascular anatomy. This demonstrates no inferiority of the proposed MxR-system. Regarding anatomical description, the MxR-group showed a tendency to perform better than the control group. Additionally, the use of MxR increased motivation without increasing the subjective load of the students.

Within the expanding field of XR-technology in medicine, the application of innovative teaching methods in medical education has gained increasing popularity. Especially in the context of surgical training and high-fidelity simulations, the role of XR seems to be rapidly growing [13, 21]. In vascular and endovascular surgery, however, XR is rarely used with regards to training and mainly concentrates on conceptual ideas [12]. So far, undergraduate training has not been addressed. To our knowledge, this is the first randomized prospective controlled study evaluating an MxR-system in the context of an educational setting in vascular surgery undergraduate education. Imaging and image-evaluation have always played a major role in vascular surgery. It seems only logical, that this image-relatedness should also be reflected in medical education. However, vascular surgery medical school curricula rarely go beyond traditional lectures and seminars [22, 23]. While several studies already addressed the application of AR/MxR-technologies in different educational contexts [12, 21], their value in vascular surgery curricula has yet to be determined. In contrast, literature on the potential of AR/MxR-technology in improving clinician’s understanding of anatomy and subsequently image-guided surgery is rapidly growing, mostly promoting this new technology as a future “game changer” [12, 24, 25]. As medicine – and especially surgery – has to find its way into a digital era [26], future health care professionals should be incorporated in this evolution at an early educational stage.

It has already been shown, that the integration of 3D-visualization technologies in anatomy classes improves learning [7, 8, 27]. This is true for both, diagnostic skills as well as visual spatial ability [8, 28]. A few studies also evaluated the application of AR/VR in anatomy teaching [27, 29]. Similar to their results, the participants of our study showed a slightly better performance in the MxR-intervention group compared to the control group. Looking at the absolute numbers, the CT-group starts at a higher level of knowledge. Regarding the diagnostic abilities in imaging analysis, though, group differences are negligible; with the exception of the anatomical subscale, in which the MxR-group showed a tendency to a better performance. Noteworthy, the mean score in imaging evaluations scored only around 63% of the maximum possible score, thereby indicating the complexity of thoracic aortic anatomy. Multiple regression analysis revealed gender and educational level of being associated with a greater learning success. Considering the role of gender, women showed an overall improvement of 1.43 points against 0.8 points in the male cohort. This could not be seen in the context of detailed image evaluation. It has been proposed that male participants are more likely to be used to XR-technologies due to a potential gaming history [30, 31]. Our findings are not strong enough to support this suspicion, given the heterogenous improvement range within the groups. Previous gaming experiences, equally been found to influence performance outcomes with respect to virtual spatial tasks [32], did not influence learning success in our study. As all participants in our study were in their 4th or 5th year of medical school, approximately 42% of them have already passed the vascular surgery curriculum, which could have influenced the test results as well. One could hypothesize, that the differences in learning success could be more evident in novices. However, studies evaluating the use of MxR- vs. CT-guided patient education showed similar levels of informational gain between the two methods [20]. Integrating a third teaching arm including actual “traditional” 2D-learning methods such as anatomical drawings, could have influenced the results. Yet, the curriculum at our medical school integrates cross-sectional imaging, e.g. contrast-enhanced cadaver specific post mortem tomography already in first-year gross anatomy teaching [5, 6]. Therefore, including an “anatomical atlas” group would have been highly artificial in our context. Regarding motivational factors, students seemed highly interested in studying with innovative technologies like MxR-systems in contrast to traditional methods. Additionally, the perceived workload was rated to be at a low level, besides the mental demand subscale, which could be attributed to a higher level of additional steps using the controller and the HMD. The mean SUS-score of 85.0 points indicates an excellent system usability [33]. This contrasts with other studies in the XR-field, in which the proposed MxR-system was rated around 57 points, displaying only marginal acceptable system usability [34]. Different systems and HMDs can differ in their usability, though.

Clearly, motivation and joy aren’t “hard facts” in traditional research. Additionally, new kids on the block have an easy time getting people excited. Thus, a technology-driven approach should not be endorsed unreservedly. Nevertheless, motivational aspects should not be neglected, especially in the context of learning, maybe even more in the context of learning in the surgical field. Facing declining interest in surgical careers in the next generation, early motivation to get in touch with the field could be a relevant pull-factor. MxR-systems have the ability to address both, motivation as well as learning outcomes. Yet, the application should be used sensibly and focus on learner-centered approaches in future research, As such, they could have the potential to increase student engagement [35, 36].

### Limitations

This study comes with several drawbacks. First, the small number of participants increases the uncertainty of the effect estimate. The implementation of the proposed teaching unit is time-consuming with approximately 3.5–4 h per participant, without considering the time for image-preparation. Given the limited number of available hardware and the currently high costs of the technical equipment, this restricts the broad application and integration into a medical school curriculum. Second, the students participating in this study were already in their 4th or 5th year of medical school, resulting in previous experience with traditional imaging techniques as mentioned above. In our institution students learn how to read a CT-scan in their very first semester, leading to a potential bias in our study. A training course of roughly 30 min cannot outweigh four years of experience.

## Conclusion

Mixed Reality represents a helpful tool for the visualization of topographic anatomy of the thoracic vascular system. The modality can increase the student’s motivation to engage more intensively and comprehensively with the learning content. Apart from making learning more enjoyable, this study did not show any statistically sustainable, objectifiable benefit of an MxR-assisted educational approach against established teaching methods. Therefore, the effective value of such a system should not be overestimated. In addition, the current high demand on resources in terms of time, costs and staff significantly prevents broad curricular use. Nevertheless, MxR-technology is suitable for addressing the cognitive, affective and - perhaps most interestingly - the psychomotor level of learning. Future studies should take a learner-centered approach to further evaluate the utility of this technology and potentially advance the development and implementation of MxR in vascular surgery training. For MxR to gain in importance, use cases must be identified in which the physical and virtual objects are equally indispensable.

## Data Availability

All data will be available on demand.
